# Therapeutic Approaches for Acute Promyelocytic Leukaemia: Moving Towards an Orally Chemotherapy-Free Era

**DOI:** 10.3389/fonc.2020.586004

**Published:** 2020-10-20

**Authors:** Zheng-Li Xu, Xiao-Jun Huang

**Affiliations:** Peking University People’s Hospital, Peking University Institute of Hematology, National Clinical Research Center for Hematologic Disease, Beijing Key Laboratory of Hematopoietic Stem Cell Transplantation, Beijing, China

**Keywords:** acute promyelocytic leukaemia, oral, chemotherapy-free, all-*trans* retinoic acid, Realgar-Indigo naturalis formula

## Abstract

The treatment of acute promyelocytic leukaemia (APL) has evolved dramatically over the past several decades, making the disease a highly curable form of acute leukaemia. The discoveries of all-*trans* retinoic acid (ATRA) and arsenic trioxide (ATO) were landmark events, leading to historic revolutions in the treatment of APL. One major change was from chemotherapy-based to chemotherapy-free treatment regimens, and the combination of ATRA plus ATO without chemotherapy has been recommended as the standard therapy for non-high-risk APL. The other major change was from the intravenous administration of medicine in the hospital to a largely home-based oral approach, which is a more cost-effective and convenient treatment model. In this review, we focus on the evolution of therapeutic approaches for APL, as well as the challenges that remain with the current approaches.

## Introduction

Acute promyelocytic leukaemia (APL) was associated with a severe bleeding tendency and an extremely poor prognosis in history (1). In the last thirty years, the therapeutic outcomes of APL have markedly improved. The combination of all-*trans* retinoic acid (ATRA) and arsenic trioxide (ATO) is reported to result in complete remission (CR) rates exceeding 90% and overall survival (OS) rates of 85–99% (2–8).

In the evolution of therapeutic approaches for APL, the first historical milestone was chemotherapy based on anthracyclines (9). The use of daunorubicin in induction therapy improved CR rate from 13 to 55% in APL (9). However, the median duration of remission remained poor, ranging from 11 to 29 months (10, 11). Subsequently, the first therapeutic results of ATRA and ATO were published in 1988–1996 (12, 13). Besides, various oral arsenic formulations were also introducted (14). These represent the three landmarks that contributed to two revolutions in the treatment of APL: from chemotherapy-based to chemotherapy-free regimens and from the intravenous administration of medicine in a hospital to a largely home-based oral approach. These evolutions have made the treatment of APL more efficient, convenient and affordable. Here, we review the significant changes in treatment approaches for APL, as well as the challenges that remain with the current approaches.

## The First Revolution: From Chemotherapy-Based to Chemotherapy-Free Regimens

The adoption of ATRA and ATO as treatments constituted a landmark in the development of targeted therapy for APL. In the 1980s, leukaemic promyelocytes were found to possess the unique capability to undergo differentiation when exposed to ATRA (12). Later, ATO was found to be capable of eradicating APL-initiating cells, resulting in a curative effect (15).

### Improved Remission Rates and Survival Outcomes With the Combination of ATRA/ATO

In the pre-ATRA period, anthracycline-based chemotherapy regimen with or without cytosine arabinoside (Ara-C) was applied in newly diagnosed APL patients. With anthracycline alone, complete remissions were in about 50–70% of patients and median duration of remission was 6.5–26 months (9, 16). With anthracycline plus Ara-C regimen, CR rates ranged from 68 to 72% (17, 18). In the chemotherapy alone era, only 35–45% of patients achieved a cure with anthracycline-based consolidation therapy (19).

When ATRA was introduced as an APL differentiation therapy, the treatment paradigm evolved to ATRA-based regimens (combined ATRA and chemotherapy). The ATRA-based regimens in the induction phase produced improved CR rates over 90% (20–22). After obtaining CR, consolidation therapy composed of chemotherapy with or without ATRA was administered, and the long-term disease-free survival (DFS) with this treatment paradigm was estimated to range from 68.5 to 85.6% (20–23).

The introduction of the ATRA/ATO combination as a synergistic therapy created new possibilities for the treatment of APL. In the initial attempt, combined ATRA/ATO for the induction of remission followed by consolidation chemotherapy resulted in a CR rate of 95.2% and a DFS rate of 100% with a median follow-up of 18 months (24). In subsequent studies, the combination of ATRA and ATO was applied as both induction and consolidation therapy, which resulted in CR rates of 89%-92% and an OS rate of 85% (2, 3).

### Optimal Place of Combined ATRA/ATO in Newly Diagnosed APL

The APML4 study combined ATRA with ATO and idarubicin in induction therapy and used ATRA-ATO as consolidation therapy in 124 patients with all-risk groups. The survival outcomes were inspiring, with 5-year OS of 94% and EFS of 90% (7). The AML17 trial included all risk groups for newly diagnosed APL and compared a chemotherapy-free ATRA-ATO treatment regimen with the standard chemotherapy-based regimen (ATRA and idarubicin). Burnett et al. showed that the CR rated were comparable between the two groups, but ATRA-ATO group was associated with a better 4-year EFS (91.0 vs. 70.0%, *P* = 0.002) (6).

In newly diagnosed non-high-risk APL patients, a randomized multi-centre trial conducted by Lo-Coco et al. in 2013 compared ATRA-ATO combination therapy with ATRA–idarubicin therapy for induction and consolidation therapy in newly diagnosed patients and showed similar CR rates (100 vs. 95%) but improved 2-year EFS in the former (97 vs. 86%) (5). In 2017, after a prolonged follow-up among the extended therapy patients, the EFS at 50 months for patients in the ATRA-ATO versus ATRA-chemotherapy arms were 97.3 vs. 80.0% (25). Based on the above results, the combined use of ATO and ATRA as a first-line treatment for non-high-risk APL has been adopted by the National Comprehensive Cancer Network (NCCN) guidelines (26) and the European Leukaemia Net (ELN) recommendations (27).

A chemotherapy-free protocol has also been studied in newly diagnosed high-risk APL patients. Researchers from the University of Texas MD Anderson Cancer Center reported a CR rate of 96% and a 5-year EFS of 81% in high-risk patients treated with ATO, ATRA plus gemtuzumab ozogamycin [GO] for induction and ATRA/ATO for consolidation therapy (8). More recently, the SWOG Cancer Research Network has applied the chemotherapy-free combination of ATRA, ATO and GO for induction therapy followed by chemotherapy included for consolidation treatment in high-risk patients. Overall, 86% of the patients achieved CR, and the 3-year EFS was 78%, with the largest sample size (N = 70) of high-risk patients (28). Currently, there is no definitely recommended treatment option for high-risk patients (26, 27).

### Reduced Chemotherapy-Related Toxicity in the ATRA/ATO Period

The synergistic activity of combined ATRA and ATO has been indicated in the treatment of APL, thereby lessening the need for chemotherapy and reducing the risk of chemotherapy-related toxicities. Specifically, such a chemotherapy-free regimen is likely to reduce the risk of myelosuppression-related complications, cardiotoxicity associated with anthracycline exposure, and secondary malignancies associated with the use of ATRA plus traditional chemotherapy (29). In a previous study, 17% cases treated with ATRA plus idarubicin developed secondary malignancies (30).

Although ATRA plus ATO was associated with more frequent increases in liver enzymes and QTc prolongation, these effects were reversible and manageable with reduced doses or the temporary discontinuation of the drug (5). We should note that chronic arsenic exposure has potential risk of causing cancer, but there is no clinical evidence.

## The Second Revolution: From Intravenous Administration to an Oral Approach

Intravenous ATO is inconvenient, involves frequent hospitalization and requires the maintenance of vascular access. Thus, an oral arsenic drug has been anticipated for a long time; this oral approach could facilitate a largely home-based protocol and further promote the quality of life of APL patients.

In China, the study of oral arsenic formulations has a long history. There are three main inorganic arsenic forms, namely, red arsenic (tetra-arsenic tetra-sulfide, As_4_S_4_, which is isolated from realgar), yellow arsenic (As_2_S_3_, also known as orpiment), and white arsenic (arsenic trioxide, As_2_O_3_, which is made by burning realgar or orpiment) (31). Beginning in the 1970s, arsenic oxide (As_2_O_3_) was used to treat APL (1). In the decades that followed, different oral arsenic formulations have been tried, and excellent outcomes in APL have been reported (32, 33).

Realgar-Indigo naturalis formula (RIF) is a drug compound containing 30 mg of realgar, 125 mg of Indigo naturalis, 50 mg of Radix salviae miltiorrhizae, 45 mg of Radix pseudostellariae, and 20 mg of garment film in one pill. Based on its clinical results (34) and the anti-APL activity *in vitro* and *in vivo* (35), RIF has been approved by the Chinese FDA and has been commercialized and commonly available in China since 2009.

### Comparable Efficacy Between RIF and Intravenous Arsenic Formulations in Newly Diagnosed APL

#### Chemotherapy Included Protocol in Consolidation Period

Huang’s group led a multi-centre, randomized controlled phase III study (APL07) comparing oral RIF (60 mg/kg) and ATO (0.16 mg/kg) in newly diagnosed APL patients as both induction and maintenance therapies from November 2007 through September 2011 (36). All patients received three courses of consolidation chemotherapy and maintenance treatment with sequential ATRA followed by either RIF or ATO for two years. With a median follow-up of 39 months, the 2-year DFS was 98.1% in the RIF group and 95.5% in the ATO group. No significant difference was noted between the RIF and ATO groups with regard to the CR rate (99.1 vs. 97.2%; *P* = 0.62). In 2016, Huang’s group updated the follow-up data and reported that the estimated 7-year EFS rates were similar between the RIF and ATO groups (93.7 vs. 89.4%, *P* = 0.37). In addition, the estimated 7-year incidences of relapse and EFS were also similar between the high-risk and non-high-risk groups (2.4 vs. 5.0%, *P* = 0.55; 91.2% vs. 91.5%, *P* = 0.74) (37).

In the treatment of paediatric APL, another randomized, multi-centre non-inferiority trial was conducted in China to determine whether intravenous ATO can be replaced by oral RIF (38). A total of 82 patients who were 16 years old or younger were randomly assigned to the ATO (n = 42) or RIF (n = 40) group. In this trial, patients received three courses of consolidation therapy containing ATRA, ATO and low-intensity chemotherapy with mitoxantrone; cytarabine was added for high-risk patients. The estimated 5-year EFS was 100% in both groups after a median 3-year follow-up.

#### RIF and ATRA Without Chemotherapy

Huang et al. conducted a single-centre pilot study to evaluate the efficacy of oral arsenic and ATRA without chemotherapy in newly diagnosed non-high-risk APL patients. A total of 20 consecutive patients were given oral arsenic RIF (60 mg/kg) and ATRA (25 mg/m^2^) as induction therapy and then RIF on a schedule of 4 weeks on and 4 weeks off and ATRA on a schedule of 2 weeks on and 2 weeks off for 7 months as post-remission therapy (39). All patients achieved haematologic complete remission after a median time of 29.5 days. The rate of complete molecular remission was 65% at 3 months and 100% at 6 months (39). This preliminary result is encouraging and provides initial evidence for the success of a largely home-based treatment protocol for the treatment of non-high-risk APL.

Subsequently, these findings were confirmed by a multi-centre, non-inferiority, open-label, randomized, controlled phase 3 trial at 14 centres in China (40). In this trial, RIF (60 mg/kg/d) or arsenic trioxide (0.15 mg/kg/d) and ATRA (25 mg/m²/d) were administered until CR was obtained. The consolidation therapy was RIF (60 mg/kg/d) or intravenous arsenic trioxide (0.15 mg/kg/d) in one 4 weeks on and 4 weeks off regimen for four cycles and ATRA (25 mg/m²/d) in one 2 weeks on and 2 weeks off regimen for seven cycles. The estimated 2-year EFS and OS were 97 vs. 94% (*P* = 0.49) and 100 vs. 94% (*P* = 0.049), respectively. This study has suggested that non-high-risk APL can be cured using oral arsenic plus ATRA without conventional chemotherapy.

Huang’s group has tried to extend the outpatient model to newly diagnosed high-risk APL patients (41). A total of 20 patients were included in a single-centre cohort study. All subjects received oral arsenic RIF (60 mg/kg/d) and ATRA (25 mg/m^2^/d) as induction therapy until CR was achieved. Hydroxyurea (Hu, 3 g/d) or Hu (3 g/d) plus cytarabine (200 mg/d) was used to reduce the leukaemia burden if patients’ WBC counts were (10–20) × 10^9^/L or over 20 × 10^9^/L before induction until the WBC count was lower than 10 × 10^9^/L. The consolidation therapy was consistent with that in non-high-risk cases (39), including RIF in a 4 weeks on and 4 weeks off regimen for four cycles and ATRA in a 2 weeks on and 2 weeks off regimen for seven cycles. With a median follow-up of 33 months, 20 patients (100%) achieved a CR after a median time of 30 days. The 3-year estimated OS and EFS were 100% and 89.4%, respectively.

### Evidence of Oral As_2_O_3_ Solution in Relapsed or Newly Diagnosed APL

More recently, the use of oral As_2_O_3_ solution in reinduction/maintenance regimens in relapsed APL, as a front-line treatment in newly diagnosed APL and in maintenance regimens after first CR have been reported by Gill’s team with encouraging outcomes (42–44).

Since 2002, Gill et al. have conducted a prospective study among APL patients who have experienced their first relapse and used protocols that involve oral As_2_O_3_ reinduction followed by As_2_O_3_ maintenance. In detail, the reinduction therapy comprised oral As_2_O_3_, ATRA and ascorbic acid (AAA) on days 1–28 and idarubicin for 5 days. After achieving CR2, patients received consolidation therapy with idarubicin. After the completion of consolidation, maintenance therapy with the AAA regimen (2 weeks every 2 months for 2 years per protocol) was administered. Finally, all 73 patients obtained CR2 after oral As_2_O_3_-based reinduction and received oral As_2_O_3_ based maintenance. The 5-year and 10-year OS rates in the cohort were 79.5 and 67.3%, respectively (42). This cohort study showed that oral As_2_O_3_-based reinduction remained effective despite previous exposures, and it was an effective maintenance therapy for patients in CR2.

Subsequently, Gill’s team brought oral As_2_O_3_ incorporation into front-line treatment in newly diagnosed APL patients. Sixty-two consecutive patients received the AAA regimen with additional daunorubicin in younger patients for induction therapy. In the comparator group, 37 subjects received similar consolidation and maintenance therapies but did not receive oral As_2_O_3_ for induction therapy. The CR rates were both 100%, and the 5-year OS rates were comparable (100 vs. 96.9%, *P* = 0.21) in the arsenic and non–arsenic induction subgroups. However, the 5-year DFS in the arsenic induction subgroup was significantly superior to that of the non–arsenic induction subgroup (100 vs. 90.5%, *P* = 0.03) (43).

Recently, the role of As_2_O_3_ in the maintenance of CR1 in APL has also been demonstrated. A total of 129 consecutive adult patients achieved CR1 with conventional induction and consolidation. Then, they underwent AAA maintenance for 2 years. At a median follow-up of 100 months, the 10-year DFS and OS were estimated to be 85 and 87%, respectively. This analysis indicated that AAA maintenance therapy is an effective choice for long-term survival in all risk categories of APL (44).

### Reduced Cost, Shorter Hospitalization, and Improved Quality of Life With Oral Forms

Jiang et al. conducted a retrospective study and compared the medical costs and length of hospital stay between oral RIF plus ATRA and intravenous ATO plus ATRA as the first-line treatment in APL patients involved in the clinical trial APL07 at Peking University People’s Hospital. The median total medical costs were significantly lower in the RIF group ($13,183.49) than in the ATO group ($24,136.98) (*P* < 0.0001). The median total length of hospital stay in the RIF group was also obviously shorter (48 days) than that in the ATO group (54 days) (*P* < 0.0001) (45). This was also consistent with the results in the paediatric cohort, with results of 67.8 vs. 43.9 days and 68.1 vs. 48.1 days for non-high-risk and high-risk patients in the ATO and RIF groups, respectively.

Oral arsenic formulations can be administered outside the hospital, which reduces the need for hospital visits, resulting in a superior quality of life than that associated with intravenous ATO (46). In our single-centre pilot study, patients received RIF plus ATRA as induction and post-remission treatment. They resumed their usual lifestyle during post-remission therapy and rated their quality of life as nearly normal on the FACT-G questionnaire (39). In the respective cohorts from Beijing, most patients in the RIF group could resume their work or study status with a relatively better quality of life during maintenance therapy after consolidation chemotherapy (45).

## Challenges and Unresolved Issues

First, there is concern that the combination of two differentiating drugs without chemotherapy might lead to an increased risk of leucocytosis and differentiation syndrome. It has been reported that 35%-47% of non-high-risk adults develop leucocytosis after chemotherapy-free induction therapy, while 24% develop leucocytosis after ATRA plus chemotherapy induction (5, 25, 39, 47). In paediatric APL patients, the incidence of leucocytosis is reported to be much higher, reaching more than 90% (48). In addition, different kinetics of WBC proliferation were observed during induction with oral arsenic plus ATRA and ATO plus ATRA. A higher WBC count was observed in the RIF group than in the ATO group after 10 days of treatment (9.22×10^9^/L vs. 4.10×10^9^/L, *P* = 0.015) (49). Currently, prompt supportive measures such as cytoreductive chemotherapy and prophylactic corticosteroids have been proven to be useful to minimize the impact of leucocytosis (47).

In addition, CNS relapse is uncommon in patients with APL treated with traditional ATRA plus chemotherapy regimens. However, there are no formal data reporting its incidence and suggesting the need for CNS prophylaxis in the chemotherapy-free era (27, 50). It is necessary to prolong the follow-up time to determine the incidence and to conduct a randomized study to identify the optimal prophylactic strategy.

Challenges remain regarding the therapeutic use of chemotherapy-free regimens for high-risk APL. As mentioned in the updated ELN guidelines, two potential options for high-risk patients are ATRA plus ATO with the addition of appropriate cytoreductive chemotherapy and conventional ATRA plus chemotherapy. The use of chemotherapy-free or minimal chemotherapy needs to be explored in more randomized trials to enable a comparison with conventional treatment in patients in separate high-risk categories.

In summary, APL used to have a high mortality rate; however, the majority of patients can be cured at present. The emergence of ATRA and arsenic represents the beginning of targeted therapy for APL, and the advent of oral arsenic formulations allows a largely home-based oral protocol. Both therapeutic revolutions have made the treatment of APL more efficient, convenient, and affordable. In the future, challenges regarding the appropriate sequence of chemotherapy-free regimens in high-risk patients and the long-term efficacy in the new era need to be addressed.

## Author Contributions 

X-JH designed the review. Z-LX and X-JH wrote the manuscript and gave final approval for the manuscript. All authors contributed to the article and approved the submitted version.

## Funding

This work was partly supported by the Foundation for Innovative Research Groups of the National Natural Science Foundation of China (Grant No. 81621001), The Key Program of National Natural Science Foundation of China (Grant No. 81530046), The National Key Research and Development Program of China (No. 2017YFA0104500) and Peking University Clinical Scientist Program (No. BMU2019LCKXJ003).

## Conflict of Interest

The authors declare that the research was conducted in the absence of any commercial or financial relationships that could be construed as a potential conflict of interest.
