# Supplementary material for: Therapeutic Approaches for Acute Promyelocytic Leukaemia: Moving Towards an Orally Chemotherapy-Free Era
Source: Front Oncol. 2020 Oct 20;10:586004. doi: 10.3389/fonc.2020.586004 (PMC7606937; doi:10.3389/fonc.2020.586004)
Supplement: Supplementary file 1 [file Table_1.docx]

**Table 1 Selected evidence of moving towards a chemotherapy-free protocol**

| **Study (ref.)** | **No.** | **Median Age** | **Non-high** | **High** | **Induction schedule** | **CR** | **Post-remission schedule** | **Follow-up**  **(mon)** | **Survival** |
| --- | --- | --- | --- | --- | --- | --- | --- | --- | --- |
| **Estey, 2006^[2]^** | **44** | **45** | **25** | **19** | **ATRA+ATO**  **(GO for high risk)** | **89%** | **Four courses of ATRA+ATO**  **(28 weeks)** | **16** | **2-year OS 86%**  **2-year DFS 86%** |
| **Ravandi,2009^[3]^** | **82** | **47(14-81)** | **56** | **26** | **ATRA+ATO**  **(GO for high risk)** | **92%** | **Four courses of ATRA+ATO**  **(28 weeks)** | **23(0.5-65.8)** | **3-year OS 85%** |
| **Lo-Coco, 2013^[5]^** | **156** | **44.6(19.1-70.2)**  **46.6(18.7-70.2)** | **156** | **0** | 1. **ATRA-ATO** 2. **ATRA-IDA** | **100%**  **95%** | **1.Four courses of ATRA+ATO**  **2.Three cycles of ATRA+IDA, ATRA+MTZ, ATRA+IDA as consolidation and MTX+6-MP as maintenance** | **34.4(0.5-55.8)** | **2-year OS 99 vs. 91%**  **2-year EFS 97 vs. 86%** |
| **Burnett,2015^[6]^** | **235** | **47 (16-77)** | **178** | **57** | 1. **ATRA-ATO**   **(GO for high-risk)**   1. **ATRA-IDA** | **94%**  **89%** | **1.Four courses of ATRA+ATO**  **2.Four cycles of ATRA+IDA, ATRA+IDA, ATRA+MTZ, ATRA+IDA as consolidation** | **30.5** | **4-year OS 93 vs. 89%**  **4-year EFS 91 vs. 70%** |
| **Abaza,2017^[8]^** | **187** | **50 (14-84)** | **133** | **54** | **ATRA+ATO**  **(GO for high risk)** | **96%** | **Four cycles of ATRA+ATO consolidation** | **47.6(2.7-159.7)** | **5-year OS 88%**  **5-year EFS 85%** |
| **Platzbecker,2017^[25]^** | **276** | **46.6(18.8-70.2)**  **46.6(18-70.3)** | **276** | **0** | 1. **ATRA-ATO** 2. **ATRA-IDA** | **100%**  **97%** | **1.Four courses of ATRA+ATO**  **2.Three cycles of ATRA+IDA, ATRA+MTZ, ATRA+IDA as consolidation and MTX+6-MP as maintenance** | **40.6(0.1-83.6)** | **4-yearOS 99.2vs.92.6%**  **4-year EFS 97.3 vs. 80%** |
| **Lancet,2020^[28]^** | **70** | **46.5(19.1-86.3)** | **0** | **70** | **ATRA+ATO+GO** | **86%** | **Consolidation: ATO for 2 cycles, ATRA+DA for 2 cycles, and GO for two cycles.**  **Maintenance: ATRA+6-MP** | **40.8** | **3-year OS 86%**  **3-year EFS 78%** |

**Table 2 Selected evidence of oral arsenic drug, moving towards largely home-based protocol**

| **Study (ref.)** | **No.** | **Median Age** | **Non-high** | **High** | **Induction schedule** | **CR** | **Post-remission schedule** | **Follow-up** | **Survival** |
| --- | --- | --- | --- | --- | --- | --- | --- | --- | --- |
| **Zhu,2013^[36]^** | **231** | **36(15-60)** | **185** | **46** | **Oral RIF+ ATRA**  **IV. ATO+ATRA** | **99.1%**  **97.2%** | **Consolidation:**  **Three sequential cycles of HA, MA, DA**  **Maintenance:**  **RIF or ATO +ATRA for 2 years** | **39 (21-64)** | **3-year OS 99.1 vs. 96.6%**  **2-year DFS 98.1 vs. 95.5%** |
| **Yang, 2018^[38]^** | **82** | **9.4 (1-16)** | **60** | **22** | **Oral RIF+ ATRA+MA**  **IV. ATO+ATRA+MA** | **100%**  **100%** | **Consolidation:**  **Three cycles of ATRA+MA, ATO or RIF, ATRA+MA+ATO or RIF**  **Maintenance: MTX+6-MP** | **36** | **5-year EFS 100vs100%** |
| **Zhu,2014^[39]^** | **20** | **35(20-58)** | **20** | **0** | **Oral RIF+ ATRA** | **100%** | **RIF on a schedule of 4 weeks on and 4 weeks off and ATRA on a schedule of 2 weeks on and 2 weeks off for 7 months** | **14(8-19)** | **EFS 100%** |
| **Zhu,2018^[40]^** | **109** | **34 (24-47)**  **36 (30-46)** | **109** | **0** | **Oral RIF+ ATRA**  **IV. ATO+ATRA** | **100%**  **94%** | **RIF or ATO on a schedule of 4 weeks**  **on and 4 weeks off and ATRA on a schedule of 2 weeks on and 2 weeks off for 7 months** | **32** | **2-year OS 100vs.94%**  **2-year EFS 97vs.94%** |
| **Zhu,2018^[41]^** | **20** | **35.5(16-61)** | **0** | **20** | **Oral RIF+ ATRA (Hu or Ara-C if needed)** | **100%** | **RIF on a schedule of 4 weeks on and 4 weeks off and ATRA on a schedule of 2 weeks on and 2 weeks off for 7 months** | **33 (16-45)** | **3-year OS 100%**  **3-year EFS 89.4%** |
| **Gill,2019^[43]^** | **99** | **52(22-85)**  **51(23-78)** | **60** | **39** | **AAA+DA (≤70)**  **ATRA+DA (≤70)** | **100%**  **100%** | **Consolidation: DA+Ara-C**  **Maintenance AAA for 2 years** | **37 (13-82)**  **52 (14-77)** | **5-year OS 100vs96.9%**  **5-year LFS 100vs90.5%** |
| **Gill,2020^[44]^** | **129** | **46(18-82)** | **94** | **35** | **AAA+DA (≤70)** | **100%** | **Consolidation: DA+Ara-C**  **Maintenance AAA for 2 years** | **100(8-215)** | **10-year OS 87%**  **10-year RFS 85%** |

**Abbreviations: AAA, oral arsenic trioxide, all-trans-retinoic acid, ascorbic acid**
